# Impact of Preparticipating Hypohydration on Cardiopulmonary Exercise Capacity in Ambitious Recreational Athletes

**DOI:** 10.3390/nu15153333

**Published:** 2023-07-27

**Authors:** Anna Strüven, Stefan Brunner, Georges Weis, Christopher Stremmel, Daniel Teupser, Jenny Schlichtiger, Korbinian Lackermair

**Affiliations:** 1 Department of Medicine I, University Hospital Munich, Ludwig Maximilian University, Marchioninistr. 15, 81377 Munich, Germany; 2 Center for Sports Medicine, University Hospital Munich, Ludwig Maximilian University, Ziemssenstraße 5, 80336 Munich, Germany; 3 Institute of Laboratory Medicine, University Hospital Ludwig Maximilian University, Marchioninistr. 15, 81377 Munich, Germany

**Keywords:** preparticipating hypohydration, exercise capacity, recreational athletes, exercise, dehydration, recreational athletes, cardiopulmonary exercise capacity

## Abstract

Background: Heat induces a thermoregulatory strain that impairs cardiopulmonary exercise capacity. The aim of the current study is to elucidate the effect of isolated dehydration on cardiopulmonary exercise capacity in a model of preparticipating hypohydration. Methods: Healthy recreational athletes underwent a standardised fluid deprivation test. Hypohydration was assessed by bioelectrical impedance analysis (BIA) and laboratory testing of electrolytes and retention parameters in the blood and urine. The participants underwent cardiopulmonary exercise testing (CPET) with a cycle ramp protocol. Each participant served as their own control undergoing CPET in a hypohydrated [HYH] and euhydrated [EUH] state. Results: Fluid deprivation caused a mild (2%) but significant reduction of body water (38.6 [36.6; 40.7] vs. 39.4 [37.4; 41.5] %; *p* < 0.01) and an increase of urine osmolality (767 [694; 839] vs. 537 [445; 629] mosm/kg; *p* < 0.01). Hypohydration was without alterations of electrolytes, serum osmolality or hematocrit. The oxygen uptake was significantly lower after hypohydration (−4.8%; *p* = 0.02 at ventilatory threshold1; −2.0%; *p* < 0.01 at maximum power), with a corresponding decrease of minute ventilation (−4% at ventilatory threshold1; *p* = 0.01, −3.3% at maximum power; *p* < 0.01). The power output was lower in hypohydration (−6.8%; *p* < 0.01 at ventilatory threshold1; −2.2%; *p* = 0.01 at maximum power). Conclusion: Isolated hypohydration causes impairment of workload as well as peak oxygen uptake in recreational athletes. Our findings might indicate an important role of hypohydration in the heat-induced reduction of exercise capacity.

## 1. Background

The majority of the most popular worldwide sports events (e.g., FIFA World Cup, Summer Olympics, Tour de France, New York Marathon) are held during summer months. Heat, especially without acclimatisation, might impair cardiopulmonary exercise capacity relevantly [1,2,3,4,5,6,7,8,9,10]. Therefore, strategies to overcome heat-induced restrictions of physical attainment are of great interest to professional as well as recreational athletes in regard to peak performance levels. However, the best strategy is discussed controversially [2,3].

The following several influencing factors appear plausible as the leading cause of exercise capacity impairment due to heat exposure: first, heat increases the thermoregulatory burden [1,2,3]. To avoid a raise in body core temperature, the skin perfusion is elevated, resulting in a reduced cardiac output accessible for the musculature. Additionally, increased skin perfusion might reduce cardiac preload and, thereby, cardiac output [1,2,3,11,12]. Secondly, a heat-induced rise in sweat rate can lead to changes in plasma osmolality and dehydration if fluid and electrolyte loss is not compensated. The reduction of circulating plasma volume might impede cardiac output and changes in osmolality; as a result, an imbalance of electrolytes and increased core temperature might deteriorate metabolic processes that form the basis of exercise capacity [1,13,14,15] and might also increase the perception of exertion [1]. The physiological adaptation process during exercise in a high-temperature environment is complex, which makes it difficult to identify and evaluate the differences in the impact of potential confounders on exercise capacity. 

Nevertheless, several studies tried to find an effective treatment of exercise capacity impairment in the heat using strategies of pre-hydration or re-hydration, nutrient supplementation or mechanical cooling devices producing conflicting findings [2,3,16]. 

Interventional studies trying to influence specific aspects under heat conditions are challenging since they seem to fall short of identifying the pathophysiological key aspects of heat-induced impairment of exercise as confounding by other aspects of the thermoregulatory process cannot be ruled out. 

Therefore, the aim of the current trial was to evaluate the effect of isolated dehydration (without thermoregulatory strain and alteration of electrolytes, osmolality or metabolism) on cardiopulmonary exercise capacity in ambitious recreational athletes. 

## 2. Materials and Methods

Self-appointed “ambitious recreational athletes” were included from the medical staff of our hospital. Inclusion criterion was willingness to participate in our study; a proof of prior exercise performance or participation in prior competitions was not required. Ambitious was defined as an average exercise time per week of at least two hours during the previous 12 months. No exclusion was made for specific sports. 

Athletes with any pre-existing medical conditions, especially of heart and lung, were excluded as well as athletes with a history of professional sports. All subjects gave their informed consent for inclusion before they participated in the study. The study was approved by the Ethics Committee of Ludwig Maximilian University Munich (LMU) (Project identification code 22-0295, date of positive vote: 13 May 22) and conducted in accordance with the declaration of Helsinki.

The athletes underwent the protocol twice with one exercise test in euhydrated [EUH] and one exercise test in hypohydrated [HYH] state. Hypohydration was achieved undergoing a standardised fluid deprivation test for 12 h as it is standard in our endocrinological department for diagnostic workup of polydipsia [2,3]. The subjects were advised to refrain from drinking or eating meals with high fluid content (>30%, e.g., soup). No further provisions were made for frequency, quantity or composition of food. Participants did not have to document their diet or undergo standardised weighing. 

The euhydrated scenario was without any regulation and participants were asked to keep their usual dietary habits. The sequence of euhydrated and hypohydrated exercise was randomised using sealed opaque envelopes (with the randomisation on the day before the first CPET was scheduled) and took place at comparable times of day for HYH and EUH state to rule out bias due to circadian rhythm. The interval between both exercise tests was between 7 and 14 days to allow regeneration; the participants were advised not to change their habits of sports/exercise between both CPETs. Participants performed an exercise test on a cycle ergometer (Cylcus 2-RBM Elektronik-Automation GmbH, Leipzig, Germany) with a ramp protocol at ambient conditions in our department (in a room with air conditioning and unchanged targets for room temperature and humidity). After a three-minute warm-up at low intensity (25 Watt—women; 50 Watt—men) CPET started with 40 Watt (women) or 60 Watt (men) with a target cadence between 60 and 70 runnings per minute (rpm). Cardiopulmonary exercise testing (CPET) was conducted until exhaustion. The slope of the ramp protocol was calculated individually in accordance with patient’s self-assessment targeting an exercise time between 8 and 10 min. No difference in the CPET protocol was made between euhydrated and hypohydrated exercise tests. We used Metalyzer 3B R3 system by Cortex Biophysik GmbH (Leipzig, Germanny) with Meta Soft Studio. The conduct of CPET was described in our previous work [17]. Briefly, Metalyzer 3B-R3 collects gas samples breath by breath. A 1-point gas calibration to adjust for ambient conditions was performed preceding every single test. Ventilation and gas samples are matched automatically by dynamic flow control. The delay is calculated with every single breath with this dynamic flow control. The individual test was stopped at maximal muscular or respiratory exhaustion at the participant’s discretion or when cadence could no longer be sustained above 60 rpm. Heart rate was recorded via ECG, ventilatory threshold (VT) 1 was determined by (1) V-Slope method, (2) EqO_2_ increase and (3) with RER < 1. VT2 was determined by (1) VE-Slope method, (2) decrease in PetCO_2_ (3) with respiratory exchange rate (RER) > 1 [17]. There was a minimum of one week between both tests. 

The expected water intake during the 12 h before CPET might be around 1–1.5 L. Taking into consideration that our healthy subjects without pre-existing condition of kidney are able to concentrate urine highly effectively, the total loss of body weight was anticipated to be far less than 1 kg in the HYH scenario. Therefore, weighing was thought to be inappropriate as a measure of dehydration, and we chose body composition as a measure of dehydration. We used Nutribox body impedance analyser (Data Input, Pöcking, Germany) according to the manufacturer’s instruction (as described in the manufacturer’s manual available online with free access on www.data-input.de, accessed on 30 June 2023) immediately prior to CPET. Briefly, participants were in relaxed horizontal position for a few minutes before measurement. Two gel electrodes were fixed on the dominant hand, and two electrodes on the dominant foot and ankle. Nutribox measurement utilizes the bioelectrical impedance vector analysis with the following three relevant parameters [18]: resistance (R) as a measure of body water, reactance (Xc) as a measure of body cell mass, i.e., cell membranes, and finally, the corresponding phase angle (PhA) as a result of electrical phase shift of alternating current [18]. 

Blood and urine specimens were taken immediately prior to CPET. Blood (15 mL) was taken in resting position from a cubital vein. Urine (10 mL) was taken from midstream urine. 

Serum and urine clinical chemistry parameters were determined on a Roche cobas 8000 analyser, urine osmolality was determined with an osmometer model 2020 (Advanced Instruments, Norwood, MA, USA) and specific weight was measured using urine test strips read with an automated system (Siemens, Munich, Germany). The study workflow is depicted in Supplemental Figure S1. 

## 3. Statistical Analysis

All data are presented as mean and 95% CI of mean. Shapiro–Wilk test was performed to test for normal distribution. Students t-test was used for comparison of continuous variables. *p*-values < 0.05 were considered statistically significant. G*Power post hoc calculation was made to assess test power as sample size calculation was not possible in our exploratory study. All statistical analyses were performed using SPSS version 23 (IBM Corp., Armonk, NY, USA).

## 4. Results

A total of 50 participants (17 women and 33 men) were included in our study. The mean age was 29.7 [27.8; 31.7] years. A body mass index of 20.2 [19.4; 21.1] kg/m² represents a cohort of ambitious recreational athletes. The mean height was 177 [174.1; 179.8] cm, with a mean weight of 71.9 [68.4; 75.5] kg. Body fat was 26.2% [20.5; 31.9%]. Body water was decreased by about 2% after fluid deprivation (39.44 [37.35; 41.53] vs. 38.63 [36.6; 40.65] %; *p* < 0.01) with a concomitant decrease of PhA (5.83 [5.62; 6.0] vs. 5.66 [5.46; 5.9]; *p* = 0.02. Changes in laboratory testing of blood and urine are depicted in Table 1 and Supplemental Table S1. Natrium (139.4 vs. 139.9 mmol/L; *p* = 0.2), Kalium (4.2 vs. 4.2 mmol/L; *p* = 0.5), magnesium (0.81 vs. 0.83 mmol/L; *p* = 0.1) and Calcium (2.4 vs. 2.4 mmol/L; *p* = 0.2) were without significant differences. Chloride showed a discrete but significant increase (102.2 vs. 103 mmol/L; *p* = 0.05). Serum osmolality was slightly increased (289 vs. 291 mosm/kg) with a statistical trend (0.06). No relevant concentration of blood was seen with an unaltered hematocrit of 42% (*p* = 0.6). Urine was adequately concentrated with increased Natrium excretion (94.4 vs. 143.6 mmol/L, *p* < 0.01), higher osmolality (537 vs. 767 mosm/kg; *p* < 0.01) and a higher specific weight (1.017 vs. 1.021 kg/L; *p* < 0.01)

Summarized, the results of laboratory testing show compensated dehydration with concentrated urine but without relevant alterations of electrolytes and osmolality. 

**Figure 1:**
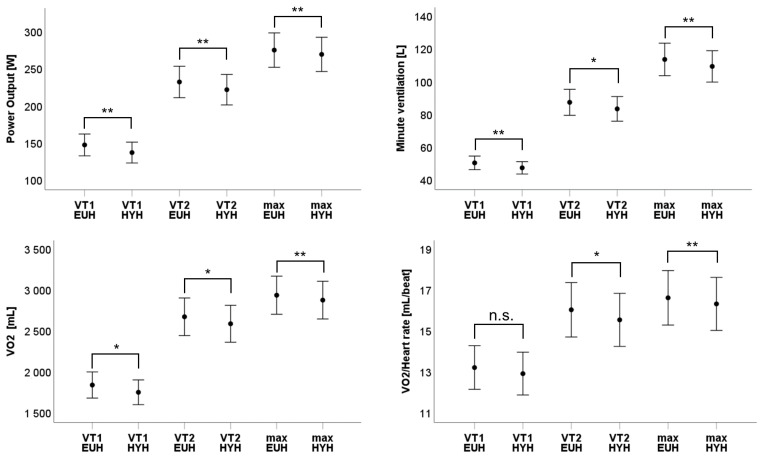
Power output [Watt), minute ventilation [litres], oxygen uptake [millilitres] and oxygen pulse/VO2/Heart rate) [millilitres] are depicted at ventilatory threshold 1 (VT1), ventilatory threshold 2 (VT2) and maximal power (max) for euhydration (EUH) and hypohydration (HYH). n.s.: not significant; *: *p* < 0.05; **: *p* < 0.01.

Table 2 and Figure 1 present the results of CPET at ventilatory threshold 1 (VT1), ventilatory threshold 2 (VT2) as well as at maximum values (VO2 max). Hypohydration was accompanied by a generalised (@ VT1, VT2 and max) and significant decrease of power output (@ VT1: −6.8%; *p* < 0.01, @ VT2: −4,7%; *p* < 0.01; @ VO2max: −2.2%; *p* = 0.01), minute ventilation (@ VT1: −4%; *p* = 0.01, @ VT2: −3.1%; *p* = 0.03; @ VO2 max: −3.3%; *p* < 0.01) and oxygen uptake (@ VT1: −4.8%; *p* = 0.02, @ VT2: −3.2%; *p* = 0.03; @ VO2 max: −2%; *p* < 0.01). The heart rate showed a delayed increase with a relevant reduction at VT1 (−2.3%; *p* = 0.04) but without differences at VT2 and maximum (@ VT2: −0.1%; *p* = 0.8; @ VO2 max: −0.4%; *p* = 0.4). Nevertheless, the oxygen pulse showed a trend at VT1 and a significant reduction at VT2 and maximum (@ VT1: −2.3%; *p* = 0.1, @ VT2: −3.1%; *p* = 0.02; @ VO2 max: −1.8%; *p* < 0.01). The reduction of minute ventilation was caused by decreased tidal volume in the beginning (@ VT1: −4.6%; *p* = 0.02) and decreased breathing rate at the end (@ VO2 max: −3.5%; *p* = 0.03) of CPET. 

No differences were seen for maximum RER (euhydrated: 1.32 [1.3; 1.35], dehydrated: 1.31 [1.28; 1.35], *p* = 0.3) and maximum lactate (euhydrated: 10.5 [9.6; 11.4], dehydrated: 10.5 [9.6; 11.4] mmol/L, *p* = 1). 

## 5. Discussion

To the best of our knowledge, this is the first trial evaluating the effect of isolated dehydration (i.e., not in the context of heat) on cardiopulmonary exercise capacity in a relevant cohort with participants of both sexes. The major findings were as follows:

- A loss of about 2 % of body water after 12 h of a standardised fluid deprivation test;
- A significant reduction of ventilation (caused by a significant reduction of tidal volume at ventilatory threshold 1 and a significant reduction of breathing rate at maximum power);
- A delayed increase in heart rate (with a significant reduction of heart rate at first ventilatory threshold) with a significant reduction of oxygen pulse (at second ventilatory threshold and maximum power);
- A resulting significant reduction of oxygen uptake and power output (as well as at both ventilatory thresholds as maximum power).

Heat decreases cardiopulmonary exercise capacity with a reduction of maximal oxygen uptake and maximal power output [1,2,3,4,5,6]. However, the effects of dehydration and thermal strain are intertwined when studying the reduction of cardiopulmonary exercise capacity in the heat. Therefore, the optimal approach to overcome this limitation of exercise capacity (i.e., reduction of thermoregulatory burden or strategies to prevent dehydration or re-hydration) remains elusive. 

To study the effects independently from a thermoregulatory burden, we simulated dehydration without a heat effect after a preparticipating fluid deprivation. 

Gonzalez-Alonso et al. studied the effect of dehydration with and without hyperthermia on ventilation in male cyclists after a prolonged intensive exercise [19]. They found hyperventilation (increased respiratory rate) under hyperthermia with dehydration compensating for reduced pulmonary blood flow. This effect could be reproduced similarly after isolated hyperthermia in contrast to isolated dehydration, which did not alter ventilation at all. 

Our findings of a significant reduction of minute ventilation (with reduced tidal volume at the beginning and a reduced breathing rate at the end of CPET) are beyond that.

The 4.6% reduction of tidal volume in the beginning CPET (@VT1) in our study might be explained by a report of Marshall et al., demonstrating a 6% reduction of forced vital capacity in dehydrated healthy athletes [20], even if the unaltered tidal volume at the end of our exercise test remains unclear. No previous data exist about respiration rate in isolated dehydration. 

Our finding of a 3.5% reduction of breathing rate at maximal power output might be interpreted in terms of reduced willingness or motivation to reach the absolute performance limit in a hypohydrated state. 

The current finding (of a delayed increase in heart rate with a significantly lower heart rate at the first ventilator threshold) contrasts with previous reports about dehydration in athletes under heat conditions. These studies have shown a faster elevation of heart rate [21]. This points out that previous findings are more an effect of thermoregulation (with a concomitant increase in dermal circulation reducing the available cardiac output for muscles) than of dehydration. The delayed increase in heart rate in hypohydration is counterintuitive and was, in addition, accompanied by a trend to reduced oxygen pulse @ VT1 that became significant @ VT2 and maximum power (at an unchanged heart rate).

Oxygen pulse can be interpreted as a measure of stroke volume in healthy individuals [22,23,24]. This reduced stroke volume should be explained with reduced cardiac preload in hypohydration. 

Besides impairment of cardiac or pulmonary capacity in hypohydrated conditions, cardiopulmonary exercise capacity assessed by CPET on a cycle ergometer could also be reduced as a consequence of reduced muscle strength. Mishull et al. studied the effect of dehydration on neuromuscular activation performance after dehydration, with a 2.1% loss in body mass in ten male subjects [4]. They found a 12% reduction in peak force with a 21% reduction in force development. Zubac et al. studied the effect of dehydration in professional boxers undergoing a rapid weight loss protocol (3%) [5]. They found a 12% reduction of force and faster exhaustion accompanied by a 53% reduction of peak lactate. Our study examined dehydration with a less pronounced loss of body weight (about 1%). Additionally, we did not find relevant differences in peak lactate. This is based on the assumption that reduced muscle strength is not the leading cause of impaired exercise capacity in our study. 

As a result of the described alterations of cardiac and pulmonary parameters, power output and oxygen uptake were significantly lower in hypohydration, which was primarily caused by a reduction of aerobic exercise capacity (−6.8% power output and 4.8% oxygen uptake at VT1). The amount of power and oxygen uptake loss was small but remarkable when considering the mild extent of dehydration (hypohydration) and the short duration of exercise. 

Our finding of dehydration-induced power loss is in line with several studies on the effects of hypohydration under heat conditions, like a study by Adams et al. [25]. In their study, 11 professional cyclists exercised at 50% of maximum oxygen uptake in the heat (30degrees centigrade). The participants received compensation for sweat-induced fluid loss intravenously or not (sham control). The power output and cycling speed were significantly lower in dehydrated subjects. 

Data about dehydration due to fluid restriction and assessment of exercise capacity without heat are sparse. The reviews state that moderate weight loss due to fluid restriction would not cause a restriction of exercise capacity [6,7]. This interpretation has to be handled with caution. Cheuvront [6] refers to five trials studying exercise capacity in a cycle ergometer setting after hypohydration due to fluid restriction but without heat [8,9,10,11,12]. The five studies included a total of only 51 participants (10, 8, 8, 8, 7). Some of these studies saw trends for a reduction of exercise capacity [8,11], but no one saw a significant reduction. It is obvious that the effects of hypohydration are easier to detect under heat conditions with a concomitant thermoregulatory burden. Nevertheless, the available negative data are more a result of lacking statistical power than proof of no effect. In contrast, our study with a cohort of 50 recreational athletes could show restrictions of pulmonary and cardio-circulatory function consistently after a very moderate loss of body weight (less than 1%).

Several studies tried to find an effective treatment of exercise capacity impairment in the heat using strategies of pre-hydration or re-hydration, nutrient supplementation or mechanical cooling devices producing conflicting findings [2,3,16]. 

Mechanical cooling seems inappropriate in a protocol like ours (as heat was absent), and re-hydration is not crucial in a short CPET (as no relevant fluid loss occurs). Although our study protocol did not include testing of treatment options, it seems obvious that maintaining preparticipating euhydration is the key factor to prevent exercise capacity impairment shown in our HYH scenario.

Limitations: Our preliminary study has the following limitations: with the used ramp protocol motivation of subjects might have influenced parameters like maximal power output or maximal oxygen uptake, as blinding to intervention was not possible. Nevertheless, the measurements of cardiopulmonary exercise capacity at the first ventilatory threshold are independent of motivation and maximal respiratory exchange rate (RER), and maximal lactate levels did not differ between the hypohydrated and euhydrated state.

The extent of dehydration was only assessed by BIA. No detailed information about fluid intake in the EUH part of the study is available. The loss of body water in BIA was not confirmed by another modality. 

There is a broad academic discussion about the role of thirst in impaired exercise capacity after dehydration [13,14,15]. Trials studying the impact of thirst are mostly using models of exercise-induced dehydration or artificial dehydration due to the use of diuretics [15]. Our model of dehydration using a preparticipating fluid deprivation test caused “isolated dehydration” without relevant alteration of electrolytes or osmolality, which are discussed to account for the perception of thirst [15]. Therefore, thirst was not within the scope of our current study. Nevertheless, we cannot rule out that our findings are biased by the perception of thirst in the HYH scenario of our study. 

Although plausible from the findings from CPET, the interpretation that reduction of cardiac preload in dehydration primarily causes reduced exercise capacity was not confirmed in other non-invasive or invasive measurements of cardiac output. 

Conclusion: mild isolated dehydration (hypohydration) causes impairment of workload as well as oxygen uptake in ambitious recreational athletes. A significant reduction of oxygen pulse indicates that this impairment is caused by reduced cardiac output as a consequence of reduced preload. Our findings of a significant reduction of exercise capacity with only mild dehydration might indicate an important role of dehydration in the heat-induced reduction of exercise capacity. 

## Figures and Tables

**Table 1:** Results of laboratory medicine testing of blood and urine for euhydration [EUH] and hypohydration [HYH]. Data are presented as mean and 95% CI of mean.

	Euhydrated [EUH]	Hypohydrated [HYH]	*p*-Value
**Blood**			
Na^+^ [mmol/L]	139.4 [138.9; 140]	139.9 [136; 144]	0.2
K^+^ [mmol/L]	4.2 [4.1: 4.3]	4.2 [4.1; 4.3]	0.5
Cl^−^ [mmol/L]	102.2 [101.6; 102.8]	103 [102.5; 103.5]	**0.05**
Mg^2+^ [mmol/L]	0.81 [0.8; 0.83]	0.83 [0.82; 0.85]	0.1
Ca^2+^ [mmol/L]	2.4 [2.4; 2.5]	2.4 [2.4; 2.4]	0.2
osmolality [mosm/kg]	289 [288; 290]	291 [290; 291]	0.06
creatinine [mg/dL]	0.94 [0.89; 1]	0.91 [0.86; 0.96]	**0.04**
hematocrit	0.42 [0.41; 0.43]	0.42 [0.41; 0.43]	0.6
**Urine**			
Na^+^ [mmol/L]	94.4 [76.3; 112.5]	143.6 [128.3; 159]	**<0.01**
osmolality [mosm/kg]	537 [445; 629]	767 [694; 839]	**<0.01**
specific weight [kg/L]	1.017 [1.016; 1.019]	1.021 [1.020; 1.023]	**<0.01**

**Table 2:** Results of CPET for euhydration [EUH], dehydration [HYH] and between group difference (∆). Data are presented as mean and 95% CI of mean at ventilatory threshold 1 (VT1), ventilatory threshold 2 (VT2) and maximum power (VO2 max).

	Euhydrated [EUH]			Hypohydrated [HYH]			Δ		
	@ VT1	@ VT2	@ V02 max	@ VT1	@ VT2	@ V02 Max	@ VT1	@ VT2	@ V02 Max
Power output [W]	148 [133; 163]	233 [212; 254]	276 [253; 299]	138 [124; 152]	222 [202; 243]	270 [247; 293]	−6.8% ***p* < 0.01**	−4.7% ***p* < 0.01**	−2.2% ***p* = 0.01**
Heart rate [min^−1^]	140 [135; 144]	167 [163; 171]	177 [174; 180]	136 [131; 141]	166 [162; 170]	176 [173; 180]	−2.3% ***p* = 0.04**	−0.1% *p* = 0.8	−0.4% *p* = 0.4
Minute ventilation [L]	50.9 [46.7; 55.1]	88.1 [80.1; 96.2]	114.4 [104.4; 124.5]	48 [44.1; 51.8]	84.1 [76.6; 91.8]	110.5 [100.9; 120]	−4% ***p* = 0.01**	−3.1% ***p* = 0.03**	−3.3% ***p* < 0.01**
VO_2_ [mL]	1858 [1697; 2019]	2695 [2463; 2927]	2958 [2723; 3193]	1769 [1616; 1922]	2609 [2381; 2837]	2900 [2667; 3131]	−4.8% ***p* = 0.02**	−3.2% ***p* = 0.03**	−2.0% ***p* < 0.01**
VO_2_ [mL^x^kg^1x^min^−1^]	25.5 [23.8; 27.3]	36.9 [34.5; 39.3]	41.1 [38.7; 43.4]	24.6 [22.8; 26.4]	36 [33.5; 38.5]	40.2 [37.9; 42.6]	−3.5% *p* = 0.07	−2.4% *p* = 0.1	−1.9% ***p* = 0.02**
VO_2_/Heart rate [mL/beat]	13.2 [12.2; 14.2]	16.1 [14.7; 17.4]	16.6 [15.3; 18]	12.9 [11.9; 14]	15.6 [14.3; 16.9]	16.3 [15; 17.6]	−2.3% *p* = 0.1	−3.1% ***p* = 0.02**	−1.8% ***p* < 0.01**
Breathing rate [min^−1^]	26 [25; 28]	35 [33; 37]	43 [40; 46]	26 [24; 27]	34 [32; 36]	42 [39; 44]	−2.4% *p* = 0.2	−2.5% *p* = 0.1	−3.5% ***p* = 0.03**
Tidal volume [mL]	1987 [1815; 2159]	2526 [2336; 2715]	2670 [2482; 2857]	1896 [1750; 2042]	2482 [2294; 2668]	2657 [2467; 2846]	−4.6% ***p* = 0.02**	−1.7% *p* = 0.3	−0.5% *p* = 0.6

## Data Availability

Data will be made available on reasonable request.

## Supplementary Materials

The following supporting information can be downloaded at: https://www.mdpi.com/article/10.3390/nu15153333/s1, Figure S1: The figure depicts the study protocol: Randomization decided starting with EUH und HYH scenario. Immediately before CPET blood and urine specimens were taken and BIA measurement was performed. Participants were placed on cycle ergometer and connected to spirometry (Resting phase). After a 3 minute warm up phase CPET started with an individualized Ramp protocol; Table S1. Results of laboratory medicine testing of blood and urine for euhydration [EUH] and hypohydration [HYH]. Data are presented as mean and 95% CI of mean; Table S2. Results of CPET for euhydration [EUH], dehydration [HYH] and between group difference (∆). Data are presented as mean and 95% CI of mean at Ventilatory Threshold 1 (VT1), Ventilatory Threshold 2 (VT2) and maximum power (VO2 max). References [26,27,28,29,30,31,32,33,34,35,36,37,38,39,40] are cited in the supplementary materials.
