# Supplementary material for: Impact of Preparticipating Hypohydration on Cardiopulmonary Exercise Capacity in Ambitious Recreational Athletes
Source: Nutrients. 2023 Jul 27;15(15):3333. doi: 10.3390/nu15153333 (PMC10421152; doi:10.3390/nu15153333)
Supplement: Supplementary file 1 [file nutrients-15-03333-s001.zip › nutrients-2453809-supplementary.pdf]

Supplemental Materials

Table S1. Results of laboratory medicine testing of blood and urine for euhydration [EUH] and hypohydration [HYH]. Data are presented as mean and 95% CI of mean

	**euhydrated**	**hypohydrated**	**G*power post hoc**
	**Blood**		
Na^+ [mmol/L]	139.4 [138.9; 140]	139.9 [136; 144]	0.33
K^+ [mmol/L]	4.2 [4.1: 4.3]	4.2 [4.1; 4.3]	0.05
Cl^- [mmol/L]	102.2 [101.6; 102.8]	103 [102.5; 103.5]	0.58
Mg^{2+} [mmol/L]	0.81 [0.8; 0.83]	0.83 [0.82; 0.85]	0.43
Ca^{2+} [mmol/L]	2.4 [2.4; 2.5]	2.4 [2.4; 2.4]	0.24
osmolarity [mmol/L]	289 [288; 290]	291 [290; 291]	0.78
creatinine [mg/dL]	0.94 [0.89; 1]	0.91 [0.86; 0.96]	0.36
hematocrit	0.42 [0.41; 0.43]	0.42 [0.41; 0.43]	0.05
**Urine**			
Na^+ [mmol/L]	94.4 [76.3; 112.5]	143.6 [128.3; 159]	0.99
osmolarity [mmol/L]	537 [445; 629]	767 [694; 839]	0.99
…specific weight	1.017 [1.016; 1.019]	1.021 [1.020; 1.023]	0.98

Table S2. Results of CPET for euhydration [EUH], dehydration [HYH] and between group difference (Δ). Data are presented as mean and 95% CI of mean at Ventilatory Threshold 1 (VT1), Ventilatory Threshold 2 (VT2) and maximum power (VO2 max).

	Euhydrated [EUH]			Hypohydrated [HYH]			G*power post hoc calculation		
	@ VT1	@ VT2	@ V02 max	@ VT1	@ VT2	@ V02 max	@ VT1	@ VT2	@ V02 max
Power Output [W]	148 [133; 163]	233 [212; 254]	276 [253; 299]	138 [124; 152]	222 [202; 243]	270 [247; 293]	0.83	0.91	0.73
Heart rate [min^{-1}]	140 [135; 144]	167 [163; 171]	177 [174; 180]	136 [131; 141]	166 [162; 170]	176 [173; 180]	0.65	0.1	0.13
Minute ventilation [L]	50.9 [46.7; 55.1]	88.1 [80.1; 96.2]	114.4 [104.4; 124.5]	48 [44.1; 51.8]	84.1 [76.6; 91.8]	110.5 [100.9; 120]	0.74	0.58	0.64
VO_2 [mL]	1858 [1697; 2019]	2695 [2463; 2927]	2958 [2723; 3193]	1769 [1616; 1922]	2609 [2381; 2837]	2900 [2667; 3131]	0.36	0.69	0.72
VO_2 [$mL*kg^{1}*min^{-1}$]	25.5 [23.8; 27.3]	36.9 [34.5; 39.3]	41.1 [38.7; 43.4]	24.6 [22.8; 26.4]	36 [33.5; 38.5]	40.2 [37.9; 42.6]	0.45	0.41	0.66
VO_2/Heart rate [mL/beat]	13.2 [12.2; 14.2]	16.1 [14.7; 17.4]	16.6 [15.3; 18]	12.9 [11.9; 14]	15.6 [14.3; 16.9]	16.3 [15; 17.6]	0.37	0.47	0.35
Breathing rate [min^{-1}]	26 [25; 28]	35 [33; 37]	43 [40; 46]	26 [24; 27]	34 [32; 36]	42 [39; 44]	0.2	0.38	0.62
Tidal volumen [mL]	1987 [1815; 2159]	2526 [2336; 2715]	2670 [2482; 2857]	1896 [1750; 2042]	2482 [2294; 2668]	2657 [2467; 2846]	0.63	0.22	0.07

Figure S1: The figure depicts the study protocol: Randomization decided starting with EUH und HYH scenario. Immediately before CPET blood and urine specimens were taken and BIA measurement was performed. Participants were placed on cycle ergometer and connected to spirometry (Resting phase). After a 3 minute warm up phase CPET started with an individualized Ramp protocol.

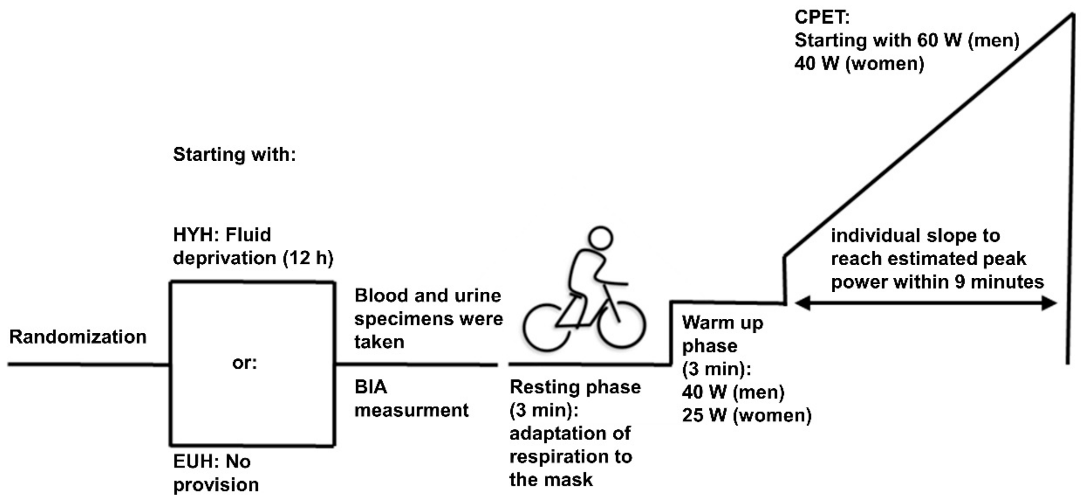

References

1. 1.Deshayes, T.A.; Pancrate, T.; Goulet, E.D.B. Impact of dehydration on perceived exertion during endurance exercise: A systematic review with meta-analysis. *J Exerc Sci Fit* **2022**, *20*, 224-235, doi:10.1016/j.jesf.2022.03.006.
2. Sjostrom, A.; Bartuseviciene, I.; Hoybye, C. Simplified and improved fluid deprivation test for diagnosing diabetes insipidus. *Eur J Endocrinol* **2021**, *184*, 123-131, doi:10.1530/EJE-20-0759.
3. Trimpou, P.; Olsson, D.S.; Ehn, O.; Ragnarsson, O. Diagnostic value of the water deprivation test in the polyuria-polydipsia syndrome. *Hormones (Athens)* **2017**, *16*, 414-422, doi:10.14310/horm.2002.1762.
4. Minshull, C.; James, L. The effects of hypohydration and fatigue on neuromuscular activation performance. *Appl Physiol Nutr Metab* **2013**, *38*, 21-26, doi:10.1139/apnm-2012-0189.
5. Zubac, D.; Simunic, B.; Buoite Stella, A.; Morrison, S.A. Neuromuscular performance after rapid weight loss in Olympic-style boxers. *Eur J Sport Sci* **2020**, *20*, 1051-1060, doi:10.1080/17461391.2019.1695954.
6. Cheuvront, S.N.; Carter, R., 3rd; Sawka, M.N. Fluid balance and endurance exercise performance. *Curr Sports Med Rep* **2003**, *2*, 202-208, doi:10.1249/00149619-200308000-00006.
7. Shirreffs, S.M. Conference on "Multidisciplinary approaches to nutritional problems". Symposium on "Performance, exercise and health". Hydration, fluids and performance. *Proc Nutr Soc* **2009**, *68*, 17-22, doi:10.1017/S002966510800877X.
8. McConell, G.K.; Burge, C.M.; Skinner, S.L.; Hargreaves, M. Influence of ingested fluid volume on physiological responses during prolonged exercise. *Acta Physiol Scand* **1997**, *160*, 149-156, doi:10.1046/j.1365-201X.1997.00139.x.
9. Fallowfield, J.L.; Williams, C.; Booth, J.; Choo, B.H.; Growns, S. Effect of water ingestion on endurance capacity during prolonged running. *J Sports Sci* **1996**, *14*, 497-502, doi:10.1080/02640419608727736.
10. Bachle, L.; Eckerson, J.; Albertson, L.; Ebersole, K.; Goodwin, J.; Petzel, D. The effect of fluid replacement on endurance performance. *J Strength Cond Res* **2001**, *15*, 217-224.
11. McConell, G.K.; Stephens, T.J.; Canny, B.J. Fluid ingestion does not influence intense 1-h exercise performance in a mild environment. *Med Sci Sports Exerc* **1999**, *31*, 386-392, doi:10.1097/00005768-199903000-00006.
12. Robinson, T.A.; Hawley, J.A.; Palmer, G.S.; Wilson, G.R.; Gray, D.A.; Noakes, T.D.; Dennis, S.C. Water ingestion does not improve 1-h cycling performance in moderate ambient temperatures. *Eur J Appl Physiol Occup Physiol* **1995**, *71*, 153-160, doi:10.1007/BF00854973.
13. Adams, J.D.; Sekiguchi, Y.; Suh, H.G.; Seal, A.D.; Sprong, C.A.; Kirkland, T.W.; Kavouras, S.A. Dehydration Impairs Cycling Performance, Independently of Thirst: A Blinded Study. *Med Sci Sports Exerc* **2018**, *50*, 1697-1703, doi:10.1249/MSS.0000000000001597.
14. Cheung, S.S.; McGarr, G.W.; Mallette, M.M.; Wallace, P.J.; Watson, C.L.; Kim, I.M.; Greenway, M.J. Separate and combined effects of dehydration and thirst sensation on exercise performance in the heat. *Scand J Med Sci Sports* **2015**, *25 Suppl 1*, 104-111, doi:10.1111/sms.12343.
15. Sawka, M.N.; Noakes, T.D. Does dehydration impair exercise performance? *Med Sci Sports Exerc* **2007**, *39*, 1209-1217, doi:10.1249/mss.0b013e318124a664.
